# Nanowire Array Breath Acetone Sensor for Diabetes Monitoring

**DOI:** 10.1002/advs.202309481

**Published:** 2024-03-13

**Authors:** Shiyu Wei, Zhe Li, Krishnan Murugappan, Ziyuan Li, Mykhaylo Lysevych, Kaushal Vora, Hark Hoe Tan, Chennupati Jagadish, Buddini I Karawdeniya, Christopher J Nolan, Antonio Tricoli, Lan Fu

**Affiliations:** ^1^ Australian Research Council Centre of Excellence for Transformative Meta‐Optical Systems Department of Electronic Materials Engineering Research School of Physics The Australian National University Canberra ACT 2600 Australia; ^2^ Commonwealth Scientific and Industrial Research Organisation (CSIRO) Mineral Resources Private Bag 10 Clayton South VIC 3169 Australia; ^3^ Nanotechnology Research Laboratory Research School of Chemistry College of Science The Australian National University Canberra ACT 2600 Australia; ^4^ Australian National Fabrication Facility The Australian National University Canberra ACT 2600 Australia; ^5^ School of Medicine and Psychology College of Health and Medicine The Australian National University Canberra ACT 2600 Australia; ^6^ Department of Diabetes and Endocrinology The Canberra Hospital Garran ACT 2605 Australia; ^7^ Nanotechnology Research Laboratory Faculty of Engineering The University of Sydney Camperdown 2006 Australia

**Keywords:** acetone sensor, breath test prototype, chitosan, Diabetic ketoacidosis, InP nanowires

## Abstract

Diabetic ketoacidosis (DKA) is a life‐threatening acute complication of diabetes characterized by the accumulation of ketone bodies in the blood. Breath acetone, a ketone, directly correlates with blood ketones. Therefore, monitoring breath acetone can significantly enhance the safety and efficacy of diabetes care. In this work, the design and fabrication of an InP/Pt/chitosan nanowire array‐based chemiresistive acetone sensor is reported. By incorporation of chitosan as a surface‐functional layer and a Pt Schottky contact for efficient charge transfer processes and photovoltaic effect, self‐powered, highly selective acetone sensing is achieved. The sensor has exhibited an ultra‐wide acetone detection range from sub‐ppb to >100 000 ppm level at room temperature, covering those in the exhaled breath from healthy individuals (300–800 ppb) to people at high risk of DKA (>75 ppm). The nanowire sensor has also been successfully integrated into a handheld breath testing prototype, the Ketowhistle, which can successfully detect different ranges of acetone concentrations in simulated breath samples. The Ketowhistle demonstrates the immediate potential for non‐invasive ketone monitoring for people living with diabetes, in particular for DKA prevention.

## Graphical abstract

The Indium Phosphide nanowire array sensor facilitated by Schottky contact and chitosan surface‐functionalization achieve self‐powered, highly selective acetone sensing at room temperature with ultra‐wide detection range (sub‐ppb to >100 000 ppm), covering the exhaled breath acetone concentration range from healthy individuals to those at high risk of ketoacidosis. This sensor is successfully integrated into a handheld breath‐testing prototype, the Ketowhistle.

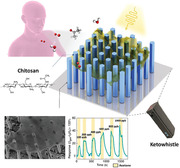

## 1 Introduction

With the global number of individuals diagnosed with diabetes mellitus cases predicted to reach 643 million by 2030, the demand for rapid, sensitive, and low‐cost health monitoring and diagnostic technologies is experiencing unprecedented growth.^[^ ^1^ ^]^ Individuals managing type 1 diabetes, as well as a rising number with type 2 diabetes, particularly those managed with sodium glucose co‐transporter inhibitor 2 (SGLT2) glucose‐lowering medications, are susceptible to diabetic ketoacidosis (DKA), a severe and life‐threatening condition.^[^ ^2^ ^]^ DKA arises from insulin deficiency, leading to the uncontrolled release of fatty acids from fat and excessive production of ketones in the liver.^[^ ^3^ ^]^ Ketones in blood are either metabolized by the body for energy or excreted from the body through urine, sweat, and exhaled breath. In urine and sweat, ketones typically manifest as 3‐β‐hydroxybutyrate (3β‐HB) and acetoacetate (AcAc), respectively, while breath predominantly contains acetone, a volatile ketone.^[^ ^4^ ^]^ Due to the seriousness of DKA, ketone monitoring is strongly recommended, particularly in situations of increased risk (e.g., in sick day management). Current clinical detection methods heavily rely on blood and urine assays, and breath ketone monitoring has not been established.^[^ ^5^ ^]^ Although finger‐prick tests using ketone strips are the most prevalent means of monitoring ketone levels, their invasive nature contributes to low levels of compliance, especially in children and younger individuals. Compounding the issue of poor compliance with blood ketone testing is the limited shelf life of the blood ketone testing strips and their associated cost. Urine testing is usually not recommended, as it is inconvenient and has lower accuracy.

Considering the severity of DKA, the escalating risk associated with the use of the SGLT2 inhibitors, and the current challenges of employing invasive ketone monitoring through blood, especially in children, there is a pressing demand for innovations in non‐invasive ketone monitoring. A swift and non‐invasive breath‐sensing methodology could offer a viable solution. It is already known that acetone concentrations in breath exhibit a direct correlation with blood ketone levels,^[^ ^6^ ^]^ making acetone in breath an ideal alternative non‐invasive biomarker for DKA detection in point‐of‐care and self‐use diagnostics. Typically, individuals with well‐managed type 1 and type 2 diabetes exhibit slightly elevated breath acetone levels compared to non‐diabetic individuals (<1 ppm) but are usually <2 ppm.^[^ ^7^ ^]^ In DKA, however, breath acetone ranges from >75 ppm to 1200 ppm.^[^ ^8^ ^]^ Overlapping with the lower end of the DKA range can occur with prolonged fasting and the use of keto‐diets for weight loss or management of epilepsy. To ensure practical applicability, the designed sensors should demonstrate a rapid response rate, high selectivity, and a sufficient lower (sub‐ppm) and upper detection limit (above 2000 ppm). These requirements are highly challenging, especially considering the high humidity, temperature fluctuations, and interference of contaminants and gases in human breath.^[^ ^6^, ^9^ ^]^ Much effort has been made to overcome these challenges to build diagnostic tools for analyzing exhaled breath samples.^[^ ^10^ ^]^ However, so far, only a few breath tests have been extended to clinical applications,^[^ ^11^ ^]^ and these applications seldom employ portable, self‐use, real‐time diagnostic devices, highlighting the demand for suitable novel technologies.

Chemiresistive gas sensing has attracted extensive attention as a promising technology for breath analysis, offering superior sensitivity, material flexibility, low power consumption, and compact size for easy integration into portable devices.^[^ ^12^ ^]^ Recent advancements include the demonstration of chemiresistive acetone sensors utilizing metal‐oxides^[^ ^13^ ^]^ and 2D materials,^[^ ^14^ ^]^ featuring ppm‐level sensitivity. In these sensors, acetone detection relies on the formation of chemisorbed oxygen species from ambient oxygen that trap electrons from the sensing materials. However, an inevitable requirement for most of these devices is the operation under high temperatures (>300 °C) or light illumination to enable the formation of the chemisorbed oxygen species,^[^ ^13^ ^]^ e.g., O_2_^−^, O^−^, and O^2−^,^[^ ^15^ ^]^ resulting in elevated power consumption and safety concerns. In addition, many reported acetone sensors also have limited selectivity, showing reactions to other volatile organic compounds (VOCs).^[^ ^16^ ^]^ Therefore, realizing highly sensitive, selective and portable acetone sensing technology operable at room temperature still appears to be a challenge.^[^ ^17^ ^]^ Our recent studies highlight InP nanowire (NW) arrays as a promising chemiresistive sensing platform for NO_2_ gas sensing with sub‐ppb level sensitivity at room temperature.^[^ ^18^ ^]^ However, from these studies, it is also noted that the InP NWs only exhibit high sensitivity specifically to NO_2_ due to the strong oxidizing nature of NO_2_, and they do not have high sensitivity to weak oxidizing or reducing gases such as CO_2_ and acetone.^[^ ^19^ ^]^

To employ the promising InP NW‐based technology for breath acetone sensing and diabetes monitoring, here we demonstrate a new NW device design, utilizing a chitosan surface‐functional layer to achieve acetone‐specific sensing. Chitosan is a cost‐effective, renewable, and eco‐friendly biopolymer^[^ ^20^ ^]^ with superb water solubility, biodegradability, renewability, antimicrobial activity, biocompatibility, and adsorption properties.^[^ ^21^ ^]^ The amine group (‐NH_2_) in chitosan interacts with the carbonyl groups of acetone, contributing to a high acetone specificity.^[^ ^22^ ^]^ Previously, chitosan has been used as the active layer of acetone sensors. However, they have shown inadequate selectivity and/or sensitivity to fulfill the clinical requirements.^[^ ^21^, ^23^ ^]^ In this work, by utilizing chitosan as the surface‐functional layer, along with a Pt Schottky contact formed on the ultrathin InP NWs, a self‐powered acetone sensor with high selectivity and sensitivity covering the whole diabetes relevant range (from sub‐ppb to >100 000 ppm level) has been achieved at room temperature. The acetone sensing mechanism was studied through numerical simulations and experimental investigations, revealing an oxygen‐facilitated two‐step charge transfer process enabling acetone redox reaction. Finally, the NW sensor has been integrated into a portable electronic setup, the Ketowhistle, serving as a prototype breath‐testing device that successfully detects simulated exhaled breath containing different ranges of acetone concentrations. It shows great promise as a low‐cost and user‐friendly breath analysis device for ketone monitoring for individuals living with diabetes and, in particular, DKA prevention.

## 2 Experimental Section

The 400 × 400 µm InP NW arrays were grown by a bottom‐up method based on the selective‐area metal‐organic chemical vapor deposition (SA‐MOCVD) technique,^[^ ^19^ ^]^ with a diameter of 50–60 nm, pitch size of 600 nm and length of ∼4 µm (details in Figure S1, Supporting Information). This diameter closely approaches the smallest one obtained from the SA‐MOCVD process. The aim of producing NWs with such a small diameter was to enhance sensitivity,^[^ ^18^ ^]^ which was investigated and proved in Section 3. The growth condition was optimized to produce thin NWs with pure wurtzite crystal structure due to dominant axial growth versus radial growth.^[^ ^24^ ^]^

**Figure 1:**
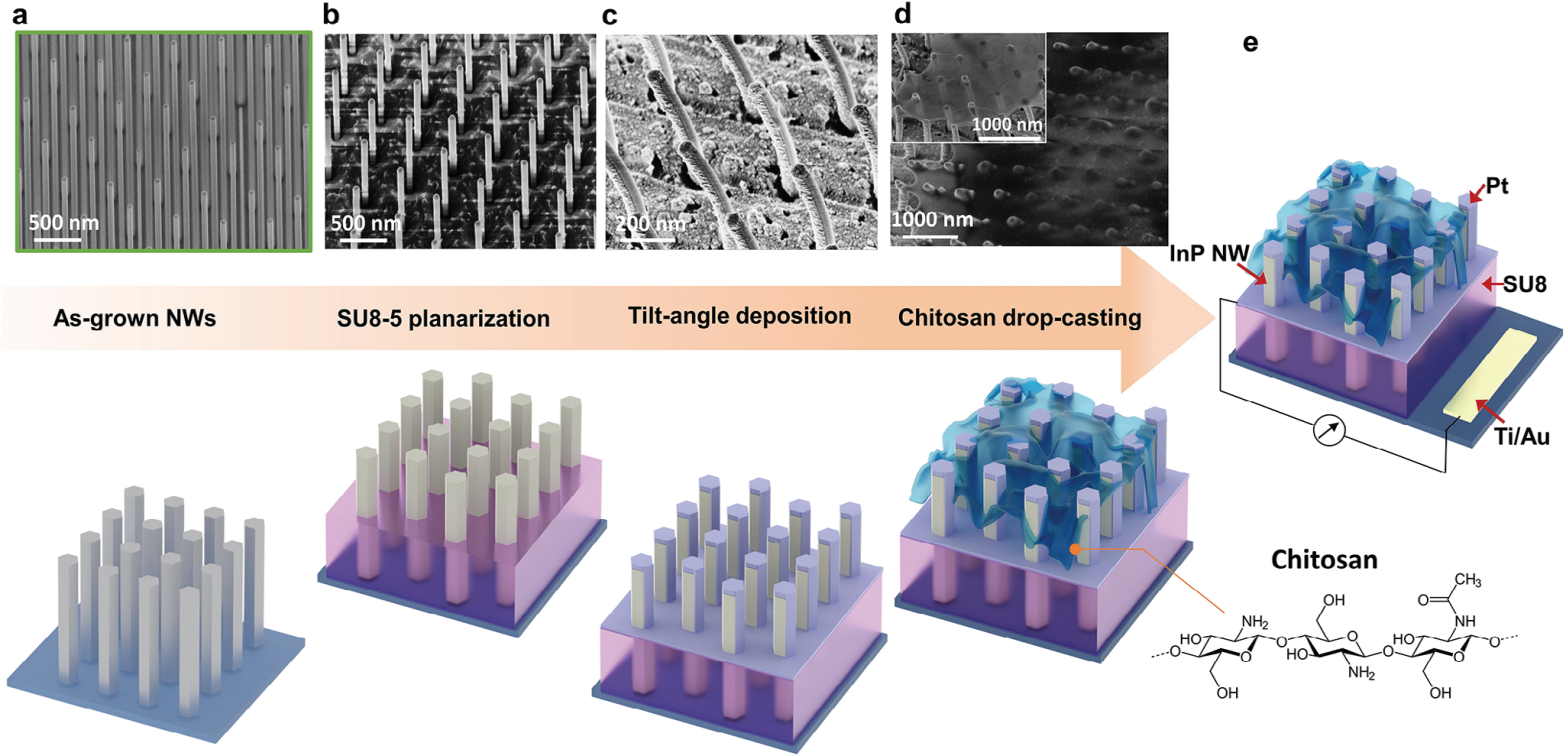
The fabrication process of the InP/Pt/chitosan NW array acetone sensor. Scanning electron microscope (SEM) images in the top panel with the corresponding schematics in the bottom panel represent the sensor device fabrication processes, including a) the as‐grown NWs; b) SU8 planarization and etching to expose the top portion of the NWs; c) tilt‐angle deposition of Pt electrode, causing a slight NW bending due to the small NW diameter; d) drop‐casting of chitosan for surface functionalization in 45° tilt‐angle imaging; and **e**, the Ti/Au bottom contact fabrication next to the NW array for electrical connection.

The as‐grown InP NW arrays were then fabricated into the acetone sensor following the process illustrated in **Figure** **1** (see Method for details). Initially, the SU8‐5 photoresist was spin‐coated to planarize the NW array, followed by oxygen plasma etching to uniformly expose the top segments of the NWs (Figure 1b). This provides mechanical stability to the thin NWs and electrical isolation between the exposed NW apexes and the substrate. The tilt‐angle deposition method^[^ ^18^ ^]^ (Figure 1c) was used to deposit a thin layer (<50 nm) of Pt on one side of the NW. This enables all the NWs in the array to be electrically connected, meanwhile allowing the Pt‐free exposed side of the NW surface to absorb light and interact with gas molecules. Subsequently, a diluted chitosan solution was drop‐casted over the NW/Pt array to form a parafilm‐like layer, functionalizing the exposed NW surface, as shown in Figure 1d. Finally, a Ti/Au contact was fabricated on top of the InP substrate next to the NW array as the bottom electrode to complete the fabrication of the acetone sensor based on bottom‐up grown NW array, i.e., the b‐InP/Pt/chitosan NW sensor (Figure 1e).

### 2.1 Methods

#### 2.1.1 Bottom‐Up Growth of InP NW Arrays by SA‐MOCVD

1. Substrate processing: At first, a 30 nm‐thick SiO_2_ layer was deposited on an n‐doped (Si) (111)A InP substrate (1–10 × 10^18^ cm^−3^) by plasma enhanced chemical vapor deposition (PECVD) at 300 °C. A negative e‐beam resist, AR6200.09, was spin‐coated (step 1: 500 rpm for 5 s; step 2: 2000 rpm for 60 s) on the SiO_2_ layer and baked at 150 °C for 1 min on the hotplate. Then, the resist was exposed to form a 400 × 400 µm hexagonal dot array pattern by a Raith 150 electron beam lithography system. After the development of resist, oxygen plasma (300 W, 2 min, 300 sccm O_2_ flow) was used to remove the resist residues in the patterned area. The exposed pattern was etched by reactive ion etching to remove the SiO_2_ layer on the InP substrate (20 sccm CHF_3_, RF power: 20 W, 4.5 min). The exposed InP surface was trim etched using 10% H_2_O_2_ (2 min) and 10% H_3_PO_4_ (2 min), repeated sequentially for 5 times. After trim etching, the sample was immediately transferred into the MOCVD reactor for NW growth.
2. InP NW growth: The InP NW arrays were grown with an AIXTRON 200/4 MOCVD reactor, operating at a base pressure of 100 mbar, using H_2_ as a carrier gas with a total flow of 14.5 L min^−1^. Trimethylindium (TMIn) and phosphine (PH_3_) were used as group III and group V precursors, respectively. Molar fractions of TMIn and PH_3_ were set at 9.38 × 10^−6^ and 7.59 × 10^−4^ mol min^−1^, respectively, corresponding to a V/III ratio of 80. All samples were hot baked at 750 °C for 10 min under a PH_3_ protective flow, and undoped InP NW (i‐InP) arrays were grown for 4 min at 730 °C. For the growth of n‐doped NW, silane (SiH_4_) was introduced, with all the other parameters kept the same as those used for the undoped InP NWs.

#### 2.1.2 Top‐Down NW Array Fabrication

The top‐down etching method for InP NW array fabrication (Extended Data Figure 1a) was reported in the previous study,^[^ ^18^ ^]^ which includes the following steps: a) PECVD deposition of ∼200 nm SiO_2_ onto an undoped (n‐type) InP wafer ((1–10) × 10^15^ cm^−3^); b) EBL patterning; c) Deposition of 70 nm Ni on the top of the EBL patterns by e‐beam evaporation followed by lift‐off in ZEP remover (Kirsten Hackenbroich) for 3 h; d) Inductively Coupled Plasma – Reactive Ion Etching (ICP‐RIE) with fluorine (ICP‐F) gas to etch the exposed SiO_2_ layer to form a Ni/SiO_2_ bilayer as the mask for subsequent InP etching; and e) InP etching with Ni/SiO_2_ mask by ICP‐RIE with chlorine gas (ICP‐CL).^[^ ^18^ ^]^

The NW diameter was determined by the mask and the ICP etching process. The smallest mask size that could be achieved was ∼40–50 nm through EBL and Ni deposition. The SEM images in Figure 1d show that the fabricated NWs exhibit large tapering due to the etching of the exposed NW sidewalls during the deep etching of the substrate, leading to a gradual change in the NW diameter from top (∼30 nm) to bottom (∼130 nm). Another limitation of the top‐down approach was the shorter NW length due to the etching selectivity of the mask and InP substrate, as the Ni/SiO_2_ mask for ICP‐RIE etching would be slowly removed during the etching process. The average length of top‐down etched NW was ∼2 µm, considerably shorter than that of the bottom‐up grown NWs (>4 µm).

#### 2.1.3 Acetone Sensor Fabrication

To fabricate a chemiresistive sensor based on the InP NW array, the SU8‐5 photoresist (Kirsten Hackenbroich) was spin‐coated to cover the entire NW array. To prevent the NW from breaking up during the fabrication process, a low spin speed of 1000 rpm was applied, followed by a two‐step baking process at 65 °C and 95 °C for 2 min each. Then, the SU8‐5 film was etched by a barrel‐etcher (PVA Tepla Gigabatch 310 m) with an O_2_ flow rate of 300 sccm and a power of 500 W to expose the top ≈500 nm of the NWs for subsequent electrical contact. Then, the sample was flood exposed under UV illumination and baked at 150 °C to solidify the SU8‐5 film. This SU8‐5 layer was used to electrically isolate the top of the NW from the InP substrate, as well as to provide mechanical support to the NWs. The E‐beam evaporator was then used to deposit a 60 nm Pt layer on the NWs for top contact, and the samples were mounted on a special holder to enable the tilted‐angle deposition. This tilted‐angle deposition method was applied to interconnect all NWs for electrical signal extraction and partially expose the NW surface for gas sensing measurement. Finally, the chitosan acetic acid aqueous solution with a concentration of 2.5% (diluted at a 1:10 ratio from the saturated solution with a concentration of 28%) was drop‐casted on the Pt‐deposited NW followed by baking on a hotplate at 50 °C to remove the solvent.

#### 2.1.4 Dark/Light Current‐Voltage Characterization

**Figure 2:**
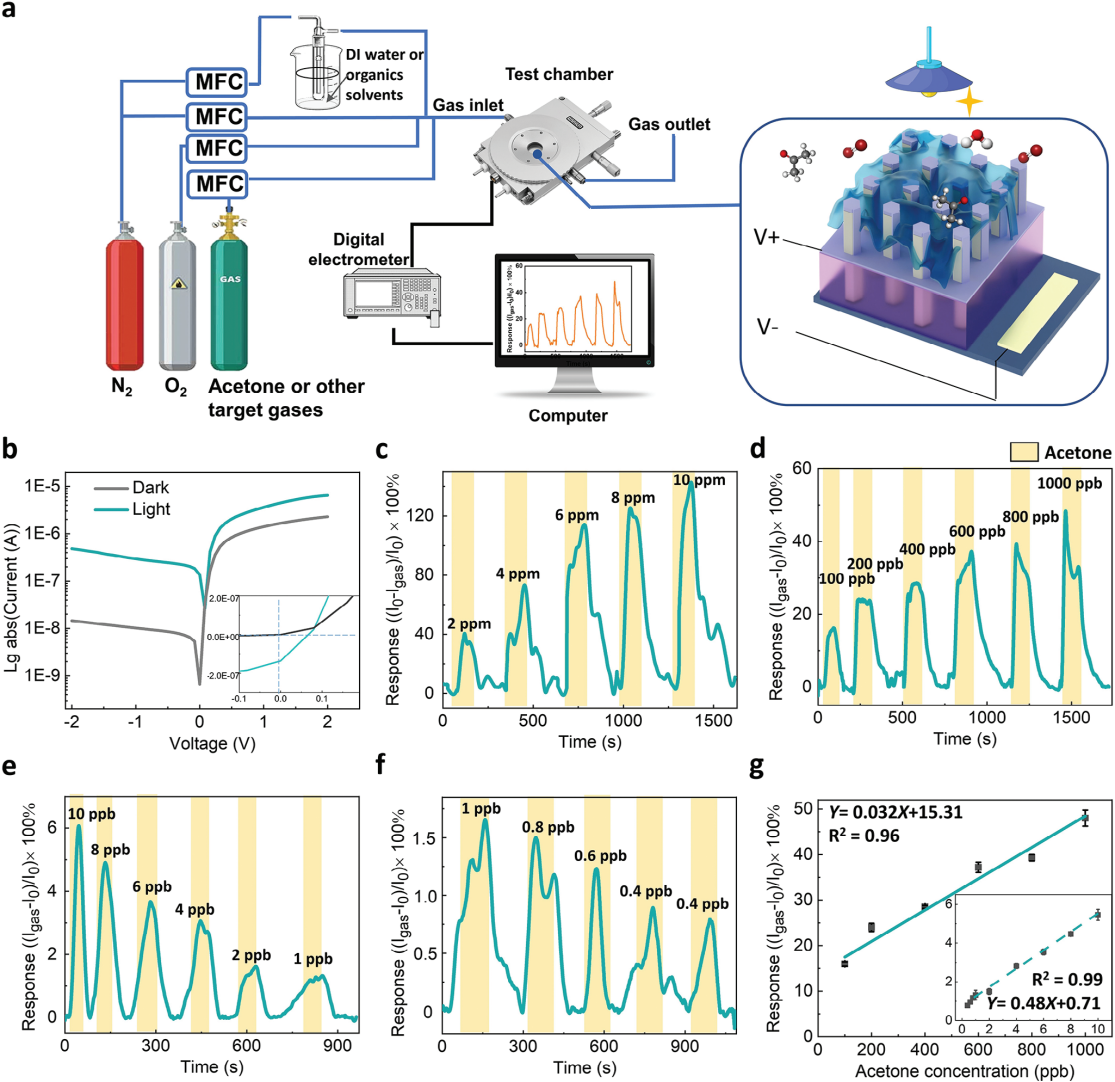
Acetone sensing performance of the b‐InP/Pt/chitosan NW sensor. a) Schematic of the laboratory gas sensing setup for acetone sensing measurement. b) *I*–*V* curves measured from the sensor device under dark/light conditions. Inset: zoom‐in plot of the *I*–*V* curve for displaying *I*_SC_ and *V*_OC_. c) Time‐dependent sensing response measured from short‐circuit current under light illumination for different ranges of acetone concentration: c) 2–10 ppm, d) 100–1000 ppb, e) 1–10 ppb, and f) 0.4–1 ppb. The yellow strips indicate acetone exposure. g) Concentration versus response curve for the acetone concentration range of 100–1000 and 0.4–10 ppb (inset), respectively.

The dark/light current‐voltage (*I*–*V*) characteristics of the InP/Pt/chitosan NW array device were characterized by a Keysight 2900 source/measure unit under 1 Sun @AM1.5 condition (solar simulator, 100 mW cm^−2^). The *I*–*V* curve of the device shows a rectification effect (**Figure** **2**a,b), indicating a typical Schottky contact formed between InP NW and Pt.^[^ ^25^ ^]^ A photovoltaic effect was observed under the light illumination from a solar simulator with a short circuit current (*I*_SC_) of 136 nA and an open circuit voltage (*V*_OC_) of 80 mV, which was the foundation of further self‐powered sensing operation^[^ ^26^ ^]^ with the *I*_SC_ acting as the self‐powered sensing signal.

#### 2.1.5 Acetone Sensing Measurements

The gas sensing performance was measured by a custom‐made sensing setup consisting of a Linkam chamber with a sample stage and Au probes, mass flow controllers (MFCs Bronkhorst), a solar simulator, and gas cylinders as shown in Figure 2a. For gas sensing measurement, simulated air with a volume ratio of N_2_ to O_2_ at 4 (V_N2_/V_O2_  ≈4; N_2_ and O_2_, BOC gas) was used as the carrier gas. The gas flow rate was controlled by MFCs, while the total gas flow rate was kept at 1 L min^−1^ for ppm level concentrations and 0.5 L min^−1^ for sub‐ppm level concentration measurements. For the measurement of analyte gas, the target VOCs (ethanol, 9.91 ppm in N_2_, Coregas; NO_2_, 10.1 ppm in N_2_, Coregas; methanol, 10 ppm in N_2_, BOC gas; acetone, 10 ppm in N_2_, BOC gas) was diluted with the simulated air to the desired concentration before being passing through the chamber.

Sensing response (*R*) quantifies the ratio of sensing signal change before and after the exposure to an analyte, which could be calculated by the following equation:

(1) $$R=\frac{\left(I_{\mathrm{gas}}-I_{0}\right)}{I_{0}}\times 100\%$$

where *I*_0_ and *I*_gas_ denote the *I*_SC_ measured in simulated air and upon acetone gas exposure, respectively.

##### Sensitivity (S)

Generally, *S* is defined as the minimum perturbation of physical parameters that would create a detectable output change, which is obtained from the slope of the response versus concentration curve (%/ppb).

##### Limitation of Detection (LOD)

LOD was defined as the lowest concentration or amount of a substance that could be reliably detected and distinguished from the background at three times the standard deviation of sensing noise (SD^2^_noise_), i.e., LOD = 3×(SD^2^_noise_/*S*).^[^ ^15^ ^]^ The SD^2^_noise_ of both devices were obtained from the standard deviation of 100 consecutive data points of the baseline.

##### Humidity Test

Water vapor was produced by the same bubbler setup as above, where the organic solvent was replaced by DI water in the bubbler. The water vapor was injected into the gas chamber with a constant N_2_ flow rate during the whole sensing process, and the relative humidity (RH) was controlled by the N_2_ flow rate, as shown in Table S1 (Supporting Information).

High‐concentration vapor was produced by a bubbler setup with a tube inlet for N_2_ to flow into the relevant solvent, evaporating saturated vapor to the gas sensing chamber (Figure 2a). To stabilize the temperature, the bubbler was placed in a water bath maintained at 25 °C during the whole sensing measurement process. The vapor concentration *C*_con_ was calculated by the following equations:^[^ ^27^ ^]^

(2) $$F_{\mathrm{output}}=\left(\frac{P}{P_{o}-P}\right)\;F_{\mathrm{carrier}}=\alpha F_{\mathrm{carrier}}$$

(3) $$C_{\mathrm{con}}=\frac{10^{6}F_{\mathrm{output}}}{F_{\mathrm{dilute}}+F_{\mathrm{carrier}}+F_{\mathrm{output}}}=\frac{10^{6}\alpha }{\left(F_{\mathrm{dilute}}/F_{\mathrm{carrier}}\right)+1+\alpha }$$

where *F*_output_, *F*_carrier_, and *F*_dilute_ are the output, carrier, and dilute flow rate of acetone vapor from the bubbler, respectively, in standard cubic centimeter per minute (sccm), and α is the vapor pickup efficiency defined as the relative ratio of the output sample flow rate to the carrier flow. *P_o_* is the outlet pressure in the bubbler, defined by *P_o_* = *P_i_* + *P*_th_, where *P_i_* is the inlet pressure to the bubbler (1.6 bar), and *P*_th_ is the thermodynamic vapor pressure of the analyte sample. When the carrier gas is completely saturated with the analyte vapor, *P*_th_ becomes the saturated vapor pressure, *P_s_*, which can be calculated from the empirical Antoine equation:^[^ ^28^ ^]^

(4) $$log_{10}\;P_{s}=A-\frac{B}{C+T}$$

where *T* is the temperature (in °C or K according to the value of C), and A, B, and C are component‐specific constants of 4.42, 1312.25, and −32.45, respectively, for acetone at 25 °C. The calculated *P_s_* of acetone is 30.6 kPa (298 K) (https://webbook.nist.gov/cgi/cbook.cgi?ID = C67641). With different dilution ratios of the simulated air, a series of high acetone concentrations from 0.57% (5700 ppm) to 16.05% (160,500 ppm) could be obtained through this setup (**Table** **1**).

**Table 1:** Parameters for calculating the acetone concentration produced from the bubbler.

*F*_carrier_ [sccm]	*F*_vapor_ [sccm]	*F*_dilution_ [sccm]	α	Vapour Conc [ppm]	Vapour Conc [%]
1000	191.21	0	0.15	160 516.63	16.05
830	158.70	170	0.15	136 966.31	13.70
660	126.20	340	0.15	112 056.53	11.21
500	95.60	500	0.15	87 261.80	8.73
330	63.10	670	0.15	59 353.75	5.94
170	32.51	830	0.15	31 482.16	3.15
130	24.86	870	0.15	24 254.26	2.43
100	19.12	900	0.15	18 762.13	1.88
66	12.62	973	0.15	12 462.51	1.25
30	5.74	970	0.15	5703.55	0.57

##### Gas Sensing Measurement under O_2_ Eliminated Condition

This condition was realized by using pure N_2_ flow flashing the gas sensing setup for more than 1 h to minimize the O_2_ residue in the gas sensing setup and set pure N_2_ as the carrier gas during the sensing test.

#### 2.1.6 Breath Test with Ketowhistle

The left panel of Figure 5c presents the sensing responses obtained from the whole range of acetone concentration of 0.1–1000 ppm, which could be fitted by the Freundlich model (Y = 57.28X^0.043^), as shown by the blue line determined by the datafitting. Exhaled breath testing by the Ketowhistle was performed using Tedlar bags (PVDF 0.6 L, Sigma‐Aldrich) with the Push/Pull Lock Valve (Figure 5a). To simulate diabetic breath, the Tedlar bags were filled with ∼50% (v) of healthy exhaled breath mixed with ∼50% of 10 ppm acetone. This volume composition was achieved by pumping in acetone gas with MFCs at 1 L min^−1^ flow rate for 18s to fill the Tedlar bag with 0.3 L acetone gas and an equal volume of exhaled breath.

## 3 Result and Discussion

### 3.1 Acetone Sensor Performance Characterization

After the sensor fabrication, the sensing measurements were performed using a custom‐made gas sensing system comprised of a Linkam chamber with a sample stage and Au probes (for contacting sample electrodes), multiple gas inlets connected with mass flow controllers, a solar simulator and gas cylinders (Figure 2a). As shown in Figure 2b, the electrical properties of the sensors were characterized by standard dark/light *I*–*V* measurements, exhibiting a typical Schottky diode characteristic. Under illumination from a solar simulator @AM1.5 at a power of 100 mW cm^−2^ (1 sun), the device demonstrated a short circuit current (*I*_SC_) of 1.4 × 10^−7^ A (an increase from <10^−9^ A in the dark condition) and an open‐circuit voltage (*V*_OC_) of 80 mV. This underscores the sensor's self‐powered operation capability. For the sensing measurement, the baseline *I*_SC_ signal was measured under 1‐sun illumination with constant airflow and zero external bias (self‐powered). Before gas injection, the sensor was placed in the chamber under light illumination and constant simulated airflow to generate steady‐state baseline *I*_SC_. As acetone gas is injected, *I*_SC_ increases and then reaches saturation. For the experiments shown below, we kept the acetone exposure time constant at (100 s) as we noted that the response reached saturation <100 s. The variation between saturated and baseline *I*_SC_ was calculated as the sensing response (R), as defined by Equation (1) in Methods. When acetone gas was turned off and airflow resumed, *I*_SC_ returned to the initial baseline current level.

The sensing performance of the b‐InP/Pt/chitosan NW sensor was investigated at low acetone concentrations ranging from 0.4 ppb to 10 ppm. As shown in Figure 2c–f, the *R* of the NW sensor exhibits a strong concentration dependence, demonstrating high sensitivity at both ppb and ppm levels. The linear fitting results in Figure 2g illustrate *R* as a function of acetone concentration with a slope that defines the device sensitivity *S* (section 2.1.5).^[^ ^29^ ^]^ The *S* in the higher concentration range (100–1000 ppb) is 0.032%/ppb, smaller than that in the 0.4–10 ppb range (0.48%/ppb). The decreased *S* at a higher concentration range results from the charge‐release saturation due to increased analyte concentration, given that there are only a finite number of active surface sites.^[^ ^30^ ^]^ Based on the linear fitting result, Y = 0.48(%ppb) X + 0.71 (Figure 2g) and SD^2^_noise,b_ = 0.028, the LOD of our acetone sensor is calculated to be 0.18 ppb (details in Section 2.1 Method). This result indicates an excellent sensitivity, which satisfies the clinical requirement (∼1 ppm) for lower‐level breath acetone detection.^[^ ^31^ ^]^ Notably, our sensor features a rapid response time (T_res_: ∼25 s) and recovery time (T_rec_: ∼39 s), as shown in Figure S2 (Supporting Information), making it promising for real‐time diagnosis. The reproducibility of the b‐InP/Pt/chitosan NW sensor was assessed by repeating 16 cycles of acetone sensing measurement at 10, 2, and 0.1 ppm (Figure S3, Supporting Information). The obtained *R* values remain relatively consistent over time for a given concentration, with a maximum standard deviation of only 5.45%, indicating excellent stability and reproducibility in self‐powered operation.

**Figure 3:**
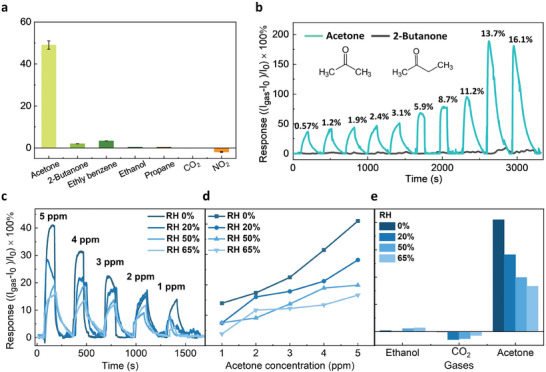
Selectivity and humidity test of the b‐InP/Pt/chitosan NW sensor. a) Selectivity measurements performed for different gases, including acetone, 2‐butanone, ethyl benzene, ethanol, propane, and NO_2_ under a concentration of 1 ppm, and CO_2_ of 1%, with the standard deviation shown as error bars derived following 10 cycles of sensing measurements. b) Time‐dependent sensing response of acetone and 2‐butanone with concentrations ranging from 0.57% (5,700 ppm) to 16.05% (160,500 ppm). c,d) Sensing response to the acetone concentration of 1–5 ppm under the RH levels of 0%, 30%, 50%, and 65%. e) Gas sensing selectivity measurement to 1 ppm acetone, ethanol, and 1% CO_2_ under different RH conditions.

Apart from the high sensitivity, achieving adequate selectivity to acetone is also critical because numerous VOCs in human breath with concentrations varying from ppt to ppm levels could act as interfering agents. A selective sensor is required to distinguish acetone from these interfering VOCs for breath ketone testing. Hence, the sensitivity of the NW sensor to a series of VOCs and atmospheric gases, including acetone (CH_3_COCH_3_), methyl nitrides (CH_3_NO_2_), ethanol (C_2_H_5_OH), propane (C_6_H_6_), was assessed at a concentration of 1 ppm. Notably, the sensor response to carbon dioxide (CO_2_), one of the main gas components in exhaled breath, was investigated at 1% (∼10 000 ppm). As shown in **Figure** **3**a, the sensing responses for these gases were less than 5% of that from acetone (49 ± 2%), indicating an excellent selectivity toward acetone. In particular, ethanol is one of the most common interfering VOCs in breath; however, the acetone selectivity over ethanol interference (*R*_acetone_/*R*_ethanol_) was >40, much higher than obtained in previous studies.^[^ ^32^ ^]^ The bubbler setup (Figure 2a) was further used to evaporate acetone and 2‐butanone solvents at concentrations ×10 000 times higher than that is used in sensing measurements in Figure 2. As shown in Figure 3b, the sensor response exhibited a consistent correlation with the concentration from 0.57% (5,700 ppm) to 13.7% (137 000 ppm). This indicates a wide dynamic range that effectively encompasses the clinically significant range, from sub‐ppm (non‐diabetic person) to 1000 ppm (DKA). It is worth noting that even though both acetone and 2‐butanone molecules are small ketones, our NW sensor only demonstrated a high sensitivity to acetone. A possible reason could be that the activation energy required for the 2‐butanone sensing reaction is higher than that required for acetone.^[^ ^33^ ^]^ This selectivity is of great significance for clinical breath analysis since 2‐butanone is a small four‐carbon ketone (acetone is a three‐carbon ketone), which is also a breath biomarker for lung cancer. The capability to distinguish it from acetone will greatly reduce the risk of false positives.^[^ ^10a^ ^]^

Another major challenge in breath analysis is the moisture in the breath, which could impact the sensor behavior by saturating the sensor and decreasing the material sensitivity to the target gas. The effect of humidity on the acetone sensing performance was characterized on b‐InP/Pt/chitosan NW devices by the same bubbler setup to create a humid condition. As shown in Figure 3c, compared to the dry air condition, the sensing response decreased by 10 ± 2%, 30 ± 6%, and 50 ± 12% under a RH of 20%, 50%, and 65%, respectively (detailed in Method and Table S1, Supporting Information). Nevertheless, according to the *R* vs concentration plots in Figure 3d, the device still maintained a consistent sensitivity *S* to acetone, indicating that the sensitivity of this device was not compromised in a humid environment. The cross‐sensitivity to interrupting molecules that may be present in exhaled breath, such as ethanol and CO_2,_ was also measured under different RH conditions separately, as shown in Figure 3e, confirming the high selectivity toward acetone, with an almost ×10 higher response compared to the interfering gases under same RH conditions.

The key performance parameters of the b‐InP/Pt/chitosan NW sensor and those of other chemiresistive acetone sensors reported in recent years are summarized in Table S2 (Supporting Information). Our NW sensor has shown superior performance in all key metrics, including sub‐ppb level detection, rapid response/recovery speed, and an ultra‐broad dynamic range covering different types of diabetes and metabolic conditions, satisfying the basic clinical requirements for breath testing. It is noteworthy that our NW acetone sensor has, for the first time, achieved self‐powered room‐temperature operation, thereby effectively addressing the power consumption and safety concerns typically encountered by most metal‐oxide based devices.

### 3.2 Investigation of the InP/Pt/chitosan NW Acetone Sensing Mechanism

**Figure 4:**
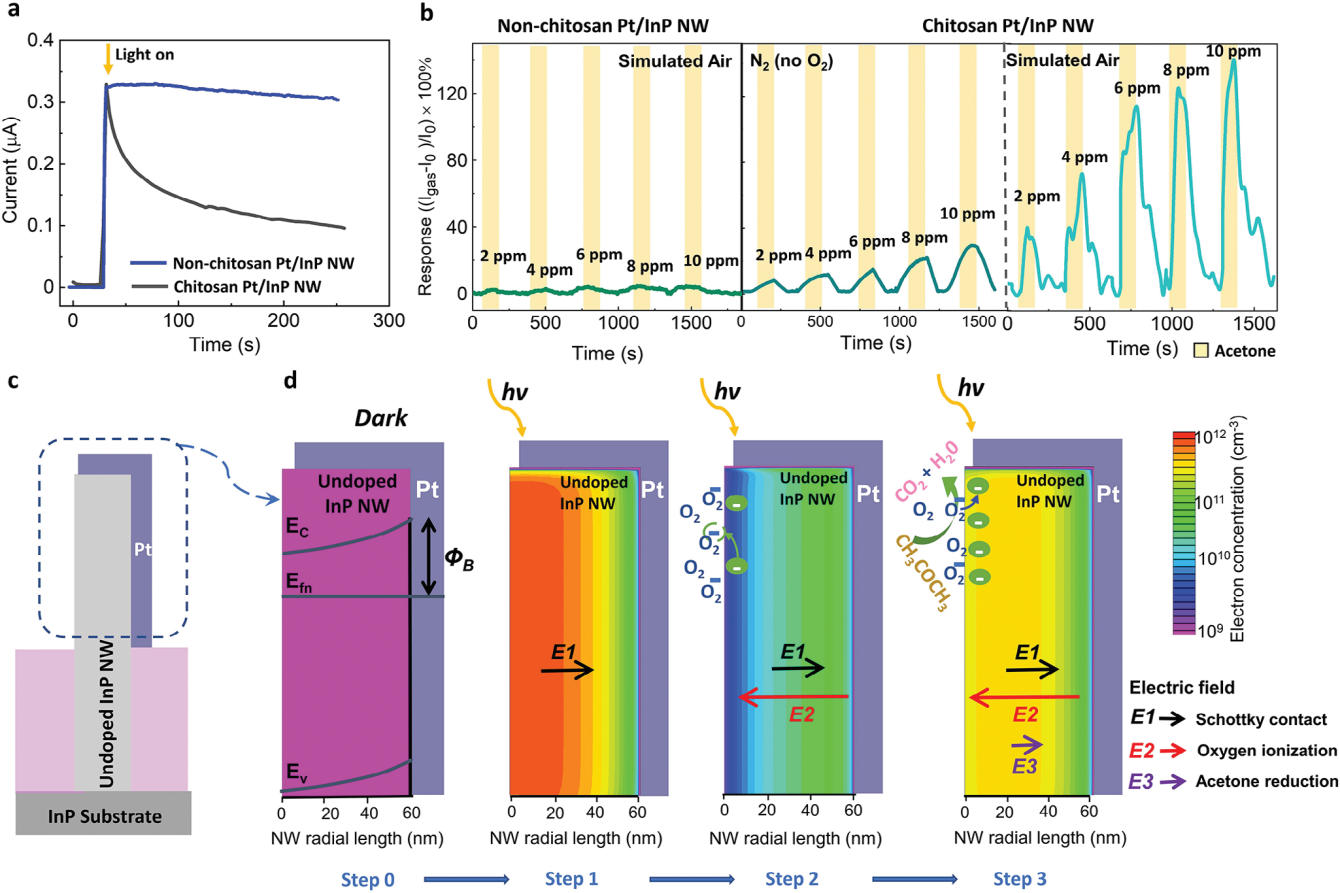
Acetone sensing mechanism investigation. a) Temporal short‐circuit current (*I*_SC_) response of the Pt/InP NW device with and without chitosan under light illumination. b) Time‐dependent acetone sensing response of the sensor with and without chitosan (under low oxygen conditions and in simulated air). c) Schematic of an InP NW with Pt contact used for the simulation study. d) Energy band diagram of the structure with the simulated electron concentration, corresponding to the various critical condition/steps of acetone sensing process. E_c_, E_v_, and E_fn_ represent the conduction band, valence band, and electron quasi‐Femi level, respectively.

To understand the sensing mechanism of the InP/Pt/chitosan NW acetone sensor, a series of controlled experiments (**Figure** **4**a,b) and simulation studies (Figure 4c,f) have been performed. Contrary to previous studies in which chitosan acts as the active sensing layer,^[^ ^21^, ^23^ ^]^ in our study, the chitosan layer was drop‐casted on top of the NW array after electrode metal deposition as the surface functionalization layer and was not electrically connected to the sensing circuit. To clarify the effect of chitosan on acetone sensing, a similar device without the chitosan layer was fabricated and used as a reference sample. Figure 4a compares the baseline *I*_SC_ of the Pt/InP NW device with/without chitosan under light illumination. Compared with the device without chitosan, the chitosan‐coated device has a significantly lower baseline current under illumination. We ascribed it to the ability of chitosan to absorb and release oxygen.^[^ ^34^ ^]^ The presence of chitosan effectively absorbs O_2_ from the simulated air (details see Method), trapping O_2_ molecules at the chitosan/InP interface, as denoted in Equation (5). This would facilitate O_2_ ionization (Equation (6)) by the photogenerated electrons that drift to the InP NW surface due to the built‐in electric field formed with the Pt/InP Schottky contact (see detailed discussion later). This depletion of electrons leads to a decreased baseline current (*I*_SC_) of the chitosan‐coated device, as shown in Figure 4a.

(5) $$O_{2(\mathrm{gas})}\;↔O_{2(\mathrm{ads})}$$

(6) $$O_{2\left(\mathrm{ads}\right)}+e^{-}\;↔{{O_{2}}^{-}}_{\left(\mathrm{ads}\right)}$$

(7) $${\mathrm{CH}}_{3}{\mathrm{COCH}}_{3\left(\mathrm{ads}\right)}+4{{O_{2}}^{-}}_{\left(\mathrm{ads}\right)}\to 3{\mathrm{CO}}_{2}+3H_{2}O+4e^{-}$$

In contrast, the device without chitosan had much less O_2_ adsorption on the InP NW surface, and thus, a negligible baseline *I*_SC_ change was observed. As the main mechanism for most chemiresistive acetone sensors, these oxygen ions are the indispensable reactant to enable a redox reaction with acetone, producing CO_2_, H_2_O and electrons,^[^ ^15^, ^16^, ^35^ ^]^ as depicted in Equation (7). Therefore, it is clear that chitosan plays a critical role in achieving sensitivity and selectivity to acetone, possibly through its ‐NH_2_ groups interacting with acetone^[^ ^21^ ^]^ and by improving O_2_ absorption. As can be seen from Figure 4b, with the simulated air, the acetone sensing response obtained from the chitosan‐coated device (>120% at 10 ppm) is significantly higher than the device without chitosan (< 5% at 10 ppm). However, under the O_2_ eliminated condition (only pure N_2_ as the carrier gas for sensing measurement; for details, see the Methods Section), the sensing response of the chitosan‐coated device decreased from > 120% (with O_2_) to < 15% (without O_2_), as shown in the middle panel of Figure 4b, confirming the vital role of the O_2(ads)_ and ionic oxygen species like O_2_^−^ in the acetone sensing reaction. The selectivity comparison of the Pt/InP NW device with/without chitosan is illustrated in Figure S4 (Supporting Information). It is clearly evident that the chitosan functionalization on InP nanowires improved the selectivity of acetone.

A simulation study by COMSOL Multiphysics was performed to investigate the effect of Pt/NW Schottky contact by calculating the energy band diagram of the device and electron concentration distribution within a single NW (Figure 4c,d). The effect of chitosan on the NW surface was also modelled through the surface charge boundary condition in the simulation. As shown in Figure 4d, under the dark condition (Step 0), the Pt layer forms a Schottky contact on the InP NW side wall. Due to the band alignment of the Fermi level between the InP NW and Pt contact, an electron depletion layer is first created that drastically decreases the electron concentration within the NW due to the small NW diameter (50–60 nm), indicating the importance of the geometric optimization. Upon light illumination, the built‐in electric field generated by the Schottky contact drives photo‐generated electrons toward the NW surface (Step 1), resulting in surface accumulation of electrons. This facilitates the ionization of adsorbed O_2_ on the NW interface (Step 2), as enhanced by chitosan functionalization and expressed in Equations (5) and (6). The enhancement of O_2_ ionization enables the acetone reduction reaction (Equation (7)), resulting in the release of electrons back to the InP NW that significantly increases the carrier concentration in the NW (Step 3).

Based on the above, it is evident that the sensing response corresponds to a two‐step electron transfer process and the resultant modulation of carrier concentration in the InP NWs. Rigorous design of the Schottky contact, NW diameter, and doping concentration is crucial to achieve the optimum acetone sensing performance, as shown and discussed in Figures S5 and S6 (Supporting Information).

### 3.3 Portable Breath Sensor Prototype (Ketowhistle) Development, Calibration, and Testing

**Figure 5:**
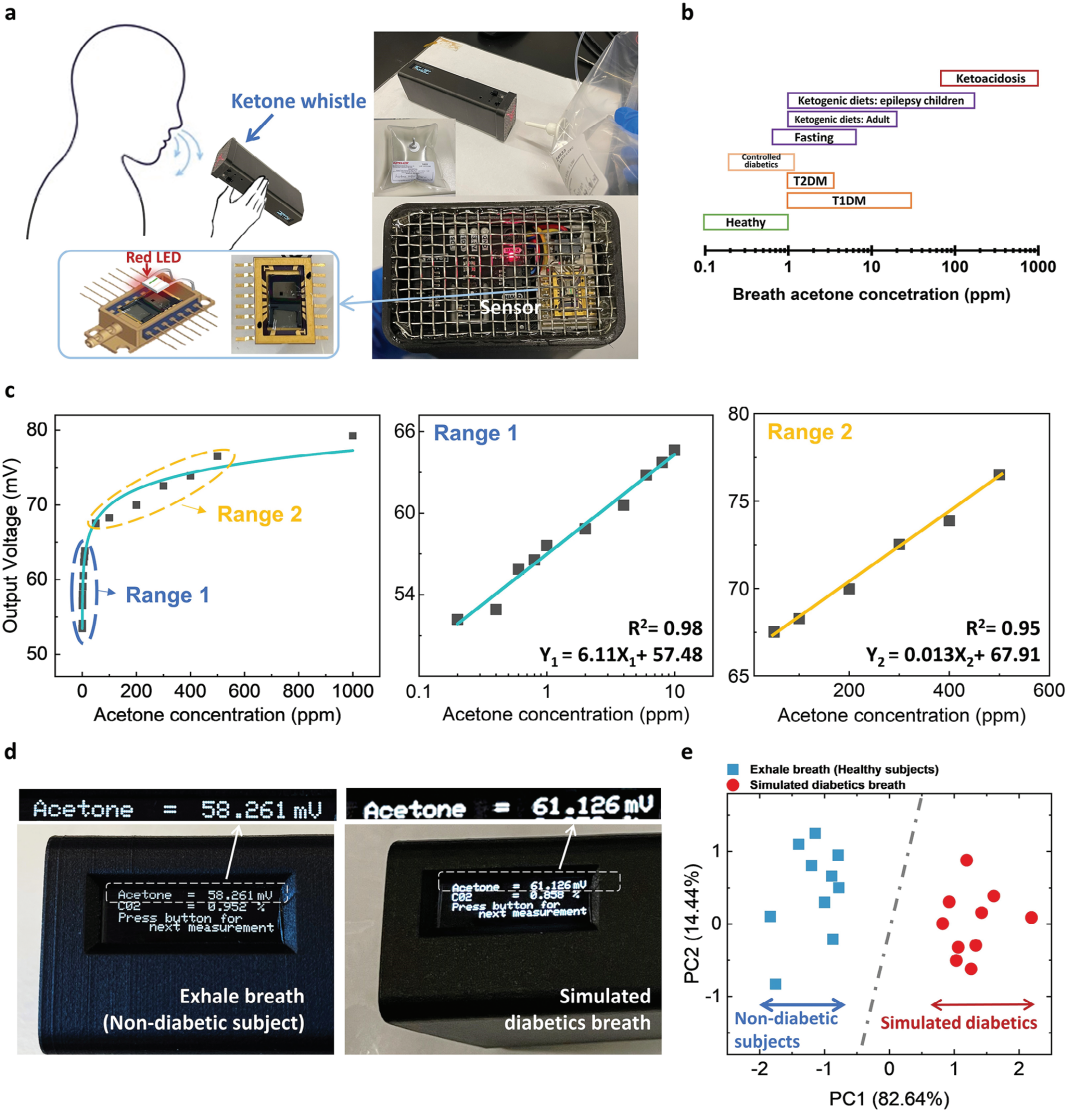
The Ketowhistle for simulated breath testing. a) Pictures of the portable Ketowhistle breath testing prototype using Tedlar bags for the collection of simulated breath samples. b) Breath acetone spectrum showing the ranges of breath acetone concentration corresponding to various physiological states and ketosis ranges.^[^ ^39c^ ^]^ c) Ketowhistle calibration for acetone concentration ranging from 0.1–1000 ppm to cover the entire breath acetone spectrum d) OLED screen displaying the response of an exhaled breath sample of a non‐diabetic (left) and a simulated diabetic breath sample (right). e) PCA for Non‐diabetic breath samples and simulated diabetic breath samples.

To demonstrate the applicability of the InP/Pt/chitosan NW array acetone sensor for point‐of‐care applications, a portable prototype device has been built for exhaled breath testing^[^ ^36^ ^]^ by integrating the NW sensor onto a handheld apparatus named Ketowhistle, as shown in **Figure** **5**a. While building the prototype device to evaluate the usability of the InP NW based acetone sensor as a handheld and portable device for DKA monitoring, we identified one drawback of using the b‐InP/Pt/chitosan sensor, the bottom‐up technology based b‐InP/Pt/chitosan NW sensor relies on specialized epitaxial growth equipment and technique, which is of high cost and difficult to scale up. Therefore, we also developed an alternative top‐down etching approach to fabricate InP NW arrays and the corresponding t‐InP/Pt/chitosan NW sensors (Figure S7, Supporting Information) with an intention for mass production and commercialization,^[^ ^37^ ^]^ as this approach is highly scalable, more accessible, cost‐effective and compatible with the well‐established complementary metal‐oxide semiconductor (CMOS) processes. The details on the device processing and sensing performance testing are provided in the Methods Section and Figure S8 (Supporting Information). Compared to the bottom‐up device, the t‐InP/Pt/chitosan NW sensor has a slightly different NW morphology (i.e., size, shape, and surface roughness). It exhibits a larger LOD of 82 ppb, shorter T_res_ (∼5 s) and T_rec_ (∼7 s), higher humidity tolerance, and ease of fabrication, despite slightly lower sensitivity, as shown in Figure S9 (Supporting Information). Considering the fabrication cost and scalability issue, the top‐down fabricated device was chosen for integration into the Ketowhistle.

The handheld Ketowhistle apparatus contains a t‐InP/Pt/chitosan NW sensor, a commercial CO_2_ sensor (to ensure the utilization of end‐tidal breath most reflective of blood acetone concentrations^[^ ^38^ ^]^), a signal‐processing circuitry, and an OLED screen for measurement display (electrical diagram provided in Figure S10 and Table S3, Supporting Information). The NW acetone sensor is illuminated by a low‐power red LED, which is placed right on top of the sensor to ensure stable illumination and sensing response, as shown in Figure 5a. Considering the wide acetone concentration range corresponding to a variety of physiological states, as summarized in Figure 5b,^[^ ^39^ ^]^ a wide concentration range calibration of the Ketowhistle for the acetone analysis was performed using the laboratory based gas sensing system. Figure 5c presents the Ketowhistle calibration for acetone concentration ranging from 0.1–1000 ppm to cover the entire breath acetone spectrum. The measured electrical signal readout from the Ketowhistle exhibits two distinct regions, with a slope of 6.11 and 0.013 mV ppm^−1^ corresponding to two ranges of acetone concentrations, i.e., Range 1 (0.1–10 ppm) and Range 2 (50–500 ppm), respectively (calibration data see Figure S11, Supporting Information). Within each range, there is a linear correlation between the electrical readout and the acetone concentration, which is consistent with the lab‐based non‐integrated device measurement in Figure 2. It is worth noting that, as indicated in Figure 5b, the low acetone concentration Range 1 covers the range that reflects normal eating patterns, whereas Range 2 indicates significant ketosis with a high risk of DKA. For persons with established diabetes, a device giving a digital readout of concentrations would be ideal in the management of sick days, as the trend in levels will indicate improvement or deterioration. These two ranges could be applied to a “traffic light” system very useful in devices used for diabetes screening (e.g., in primary care clinics) with 0.1–10 ppm shown as green (indicative of low risk of DKA), 50 to 500+ ppm shown as red (indicative of high risk of DKA), and 10–49 ppm shown as yellow (a need for caution, there may a risk of progressing to DKA). Also, the approximate linear correlation of the output voltage signal with a wide range of acetone concentration allows this device to be applied beyond diabetes management, such as monitoring ketogenic diets in weight loss plans and nutrient status in athletes.^[^ ^40^ ^]^ Although ketones are not monitored routinely in women with GDM, there is concern about the risk of ketosis affecting pregnancy outcomes if carbohydrates are too severely restricted. Thus, a simple‐to‐use device for ketone monitoring, such as the Ketowhistle, may also have a future role in pregnancy care.

For the exhaled breath test, Tedlar bags (PVDF 0.6 L, Sigma‐Aldrich), suitable for VOC sampling, were used to collect the breath samples from non‐diabetic subjects and simulated diabetic breath,^[^ ^41^ ^]^ as shown by the inset in Figure 5a. According to Figure 5b, exhaled breath in simulated diabetic breath contains acetone gas with a concentration of 1.5–20 ppm. For the exhaled breath test, the concentration of simulated breath of diabetics was adjusted to ∼5 ppm by filling the Tedlar bags with ∼50% (v) of non‐diabetic exhaled breath and ∼50% of 10 ppm acetone in simulated air. The measurement result captured by the Ketowhistle is displayed on an OLED screen, as shown in Figure 5d. Principal component analysis (PCA) was applied to analyze the output voltage‐dependent breath test results in Figure 5e,^[^ ^42^ ^]^ where the 10 simulated diabetic breath samples and 10 non‐diabetic subjects were clearly categorized into two distinguishable clusters without any overlap. The stability and reproducibility of the Ketowhistle prototype have been further demonstrated by repeated breath tests from the same person for two months, and the standard deviation is only 0.375 mV, variation <0.6% (Figure S12, Supporting Information), indicating excellent consistency essential for clinical applications. Considering the possible impact of the high moisture content in breath, the RH of the breath sample was measured along with acetone by a digital RH sensor (Figure S13, Supporting Information), indicating a minor variation between each measurement. The results from the Ketowhistle exhibit good consistency for both non‐diabatic and simulated diabetic breath samples despite the high RH level of 80–92%. The average values for these two groups of results are 58.59 ± 0.054 mV (non‐diabetic subject) and 63.60 ± 0.049 mV (diabetic subject), indicating that the Ketowhistle is able to distinguish diabetic breath from non‐diabetic breath even under high humidity conditions.

For future work, the next step is for the InP/Pt/chitosan NW sensor based Ketowhistle prototype to be assessed in pilot clinical studies, including persons living with type 1 (well and during episodes of DKA), type 2 diabetes (including those using SGLT2 inhibitor medications), and overweight/obese persons using ketogenic diets in weight loss programs. To enhance device performance, the chitosan functionalization layer can be further modified to optimize NW coverage by changing its concentration (by dilution) and/or composition.^[^ ^20^, ^34^ ^]^ Guided by this study, using various functionalization materials with functional groups specific to other breath VOCs could realize a variety of NW array‐based multiplexed breath sensor devices.

## 4 Conclusion

In this work, a novel, high‐performance, non‐invasive, self‐powered InP/Pt/chitosan NW acetone sensor has been successfully demonstrated for the first time. The sensor device exhibits superior sensitivity with an ultra‐wide acetone sensing dynamic range from sub‐ppb level up to >110 000 ppm, as well as high selectivity against other VOCs, including other small gaseous ketones. The sensing mechanism of this device is thoroughly investigated by controlled experiments and numerical simulations, revealing an oxygen‐facilitated two‐step charge transfer process for acetone reduction enabled by chitosan functionalization and Pt/InP Schottky contact. Finally, by incorporating the NW sensor into a portable breath testing prototype, the Ketowhistle, we showed their immediate potential for non‐invasive ketone testing and monitoring for persons with diabetes, particularly for DKA prevention. This study also provides an exciting pathway toward future reliable breath analysis for diabetes diagnosis, management and beyond.

## Conflict of Interest

The authors have filed a provisional patent for this work.

## Supporting information

Supporting Information

## Data Availability

The data that support the findings of this study are available from the corresponding author upon reasonable request.
