# Supplementary material for: Nanowire Array Breath Acetone Sensor for Diabetes Monitoring
Source: Adv Sci (Weinh). 2024 Mar 13;11(19):2309481. doi: 10.1002/advs.202309481 (PMC11109654; doi:10.1002/advs.202309481)
Supplement: Supplementary file 1 — Supporting Information [file ADVS-11-2309481-s001.pdf]

ADVANCED SCIENCE
Open Access

Supporting Information

for *Adv. Sci.*, DOI 10.1002/advs.202309481

Nanowire Array Breath Acetone Sensor for Diabetes Monitoring

Shiyu Wei, Zhe Li, Krishnan Murugappan, Ziyuan Li, Mykhaylo Lysevych, Kaushal Vora, Hark Hoe Tan, Chennupati Jagadish, Buddini I Karawdeniya, Christopher J Nolan, Antonio Tricoli* and Lan Fu**

Supporting Information

Nanowire Array Breath Acetone Sensor for Diabetes Monitoring

Shiyu Wei, Zhe Li, Krishnan Murugappan, Ziyuan Li, Fanlu Zhang, Mykhaylo Lysevych, Hark Hoe Tan, Chennupati Jagadish, Buddini. I Karawdeniya, Christopher Nolan, Antonio Tricoli*, and Lan Fu**

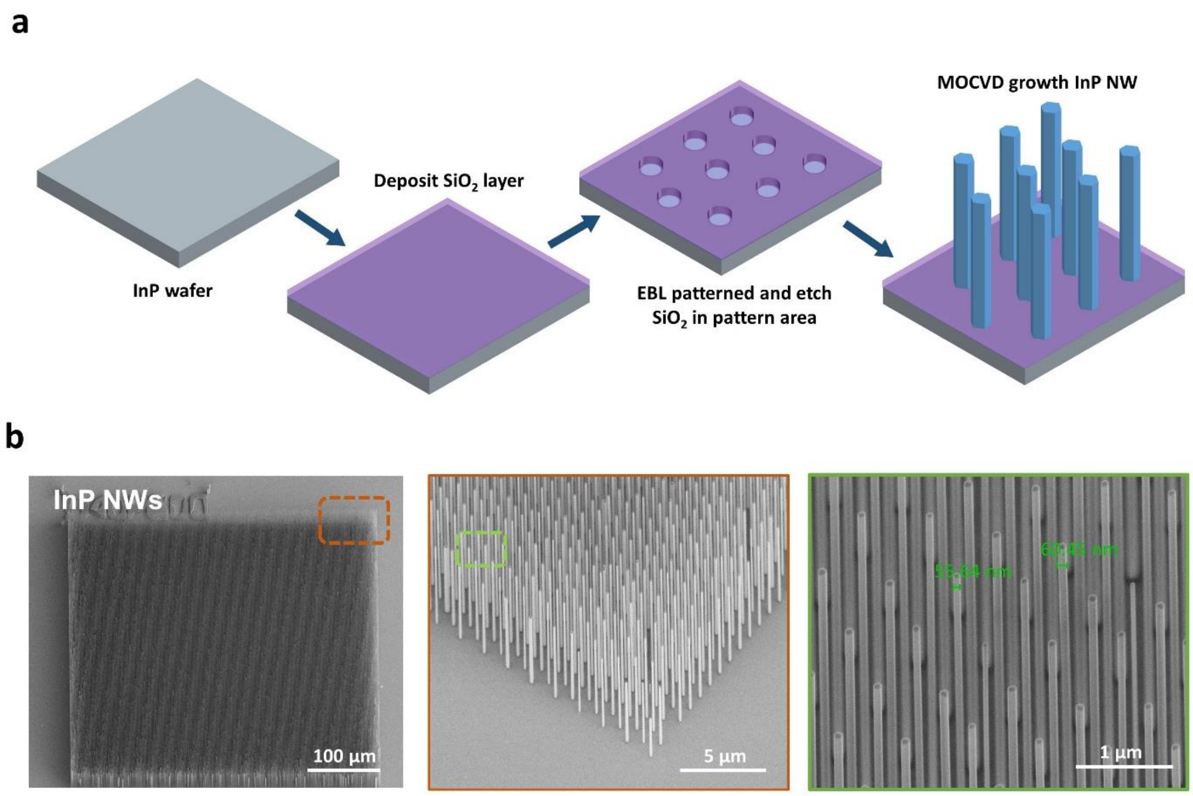

Figure S1. **a**, Schematics of selective area metal-organic chemical vapor deposition (SA-MOCVD) nanowire (NW) array growth. This method starts with substrate preparation process including the deposition of a 30 nm SiO_2 layer on InP substrate followed by electron beam lithography (EBL) patterning and reactive ion etching to form the hexagonal dot array pattern for NW array growth. **b**, The scanning electron microscope (SEM) images of the SA-MOCVD grown InP NW arrays under different magnification to characterize the overall NWs array morphology, detailed NW structure imaging (orange box) and diameter measurement (green box).

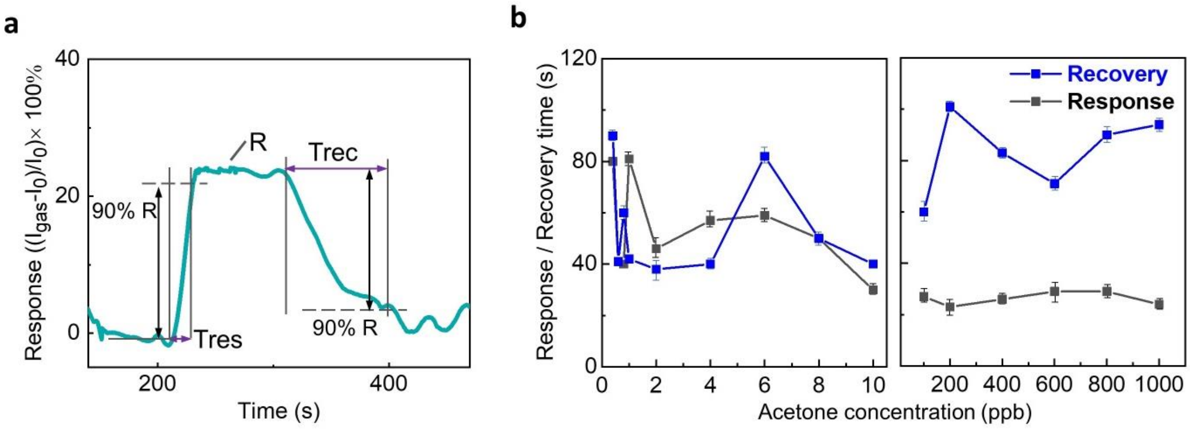

Figure S2. **a**, Calculation of the response and recovery time from the time dependent acetone sensing measurement at 200 ppb concentration. **b**, The calculated acetone sensing response and recovery time corresponding to the acetone concentration range of 0.4-10 ppb and 100-1000 ppb in Fig 2d, e, with the standard deviation as the error bars obtained by 10 cycles of sensing measurements.

The response time of the acetone sensor shows a decrease with acetone concentration from ~80 s (1 ppb) to ~20 s (100 - 1000 ppb) due to much increased adsorption rate on the NW surface and the faster concentration equilibrium established under high acetone concentration.

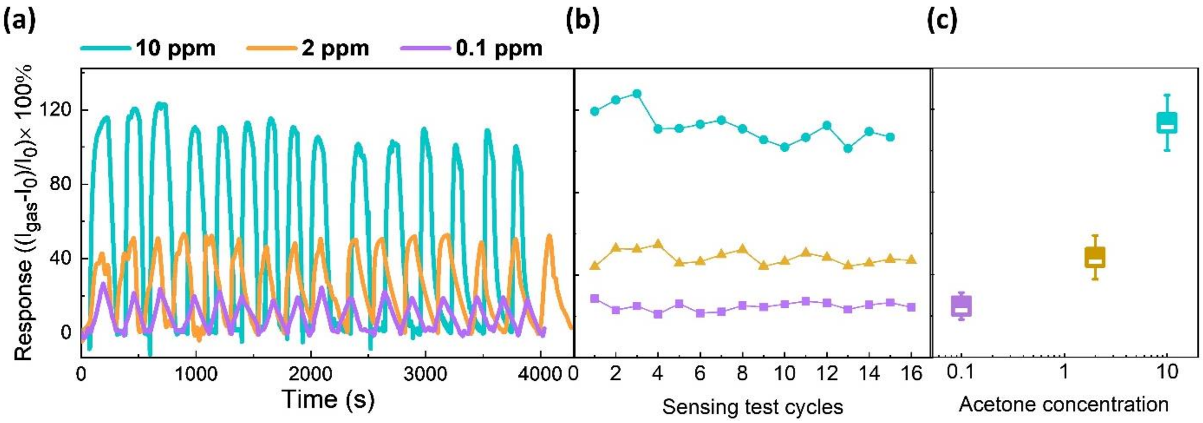

Figure S3. (a) The reproducibility measurement of b-chitosan/Pt/InP NW sensor by repeating 16 cycles of the acetone sensing measurement. (b) The sensing response vs testing cycles curve and (c) the corresponding averaged result with standard deviation.

Table S1. The humidity test at different RH controlled by flow rates of the mass flow controller

RH (%)	N_2 to the bubbler ($L \cdot min^{-1}$)	Simulated air ($L \cdot min^{-1}$)
20	0.2	0.8
50	0.5	0.5
65	0.65	0.35

Table S2. Comparison of sensing performance of different chemiresistive acetone sensors reported in recent literature.

Materials	Working temperature (°C)	Response (concentration)	Limit of detection	Response/ recover time (s)	Ref
B-InP/Pt/chitosan NW	25	48% (1 ppm)	0.4 ppb	25 / 39	This work
t-InP/Pt/chitosan NW	25	6.5% (1 ppm)	82 ppb	5 / 6	
$NiO/NiWO_4/WO_3$ (p–p–n) nanowire	400	29 (30 ppm)	0.7 ppm	-	[1]
Pt/WO_3 hemitubes	350	4.11 (2 ppm)	120 ppb	-	[2]
Si/WO_3	135	5.4 (1 ppm)	50 ppb	-	[3]
Rh/SnO_2 nanofibers	200	2.7 (1 ppm)	-	2 / 64	[4]
chitosan–Pt SnO_2 nanofibers	350	2.9 (5 ppb)	5 ppb	12 / 84	[5]
Au- ZnO/Ag core-shell films	150	43% (0.5 ppm)	0.5 ppm	45 / 160	[6]
Na/ZnO Nanoflowers	25, (UV light, 5 $mW·cm^{-2}$)	1.51 (1 ppm)	0.2 ppm	18 / 63	[7]
Zn_3N_2 / ZnO	200	5.1 (1 ppm)	0.07 ppm	15 / 27	[8]
In_2O_3 fibers	275	24 (1ppm)	0.3 ppm	-	[9]
poly[(9,9-dioctylfluorenyl-2,7-diyl)-co-(4,4-(N-(4-sec-butylphenyl) diphenylamine)]	25	13.3% (1 ppm)	0.3 ppm	-	[10]
Chitosan thin film	25	12.3% (1 ppm)	0.1 ppm	-	[11]

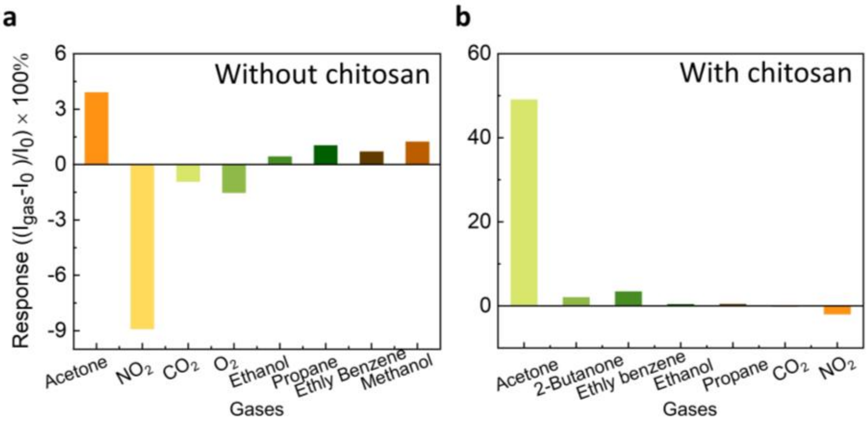

Figure S4. Selectivity measurements performed based on Pt/InP nanowire acetone sensor (a) without, (b) with chitosan for different gases, including acetone, NO_2, ethyl benzene, ethanol, propane, and methanol under a concentration of 1 ppm, and CO_2 of 1%.

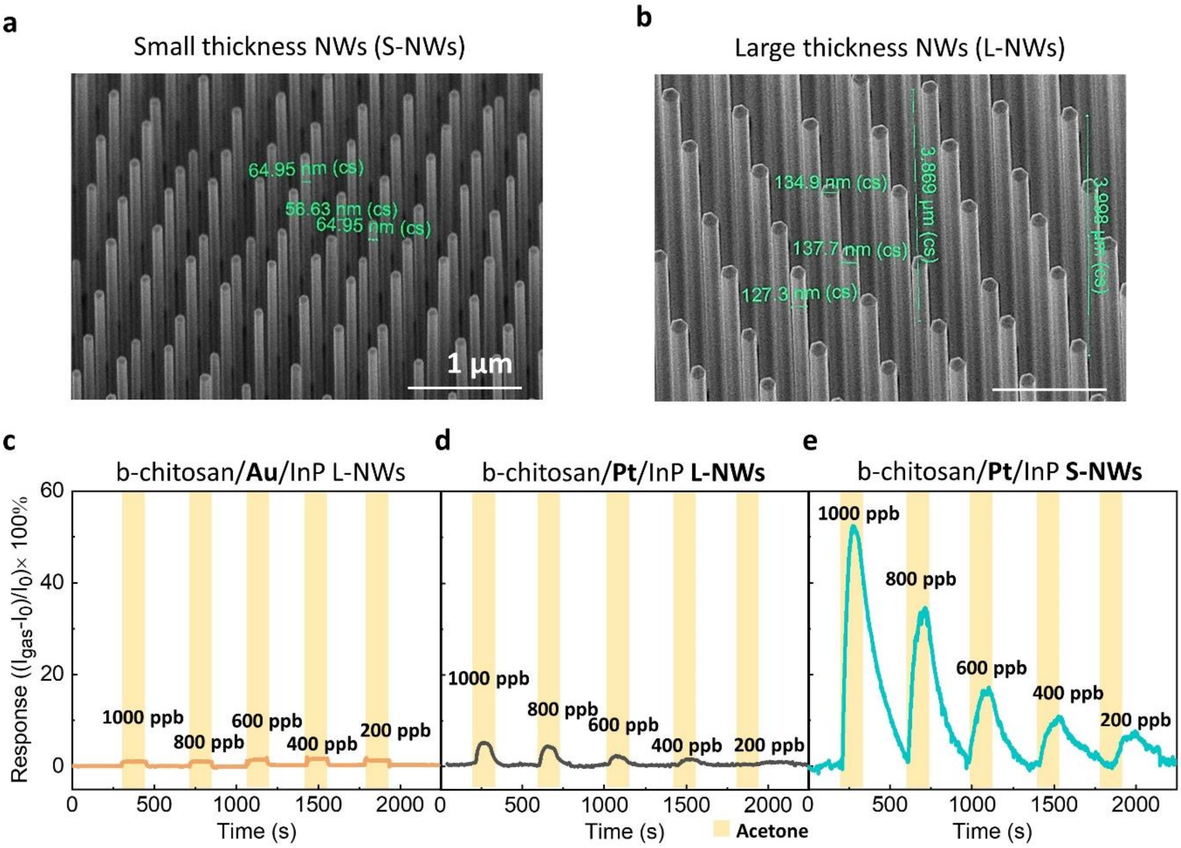

Figure S5. **a**, **b**, SEM image of small (50-60 nm) and large (120-140 nm) diameter NW array by bottom-up SA-MOVPE techniques, respectively. The time-dependent sensing response measured from: **c**, large diameter NW array sensor with Au electrode, i.e., b-InP/Au/chitosan L-NWs; **d** large diameter NW array sensor with Pt electrode, i.e., b-InP/Pt/chitosan L-NWs; **e**, small diameter NW array sensor with Pt electrode, i.e., b-InP/Pt/chitosan S-NWs.

To investigate the effect of NW diameter on acetone sensitivity, a larger diameter NW array was grown (Fig. S5b).[26] As shown in Fig. S5c and d, the devices only produced a small response (< 10 %) to acetone with a concentration of up to 1000 ppb. By reducing the NW diameter to ~50-60 nm, a significant sensitivity enhancement was observed (Fig. S5e). As Pt was replaced by Au contact, which has a smaller work function, the effectiveness of the Schottky contact was much reduced, causing a much-increased baseline current and a non-specific response to acetone (Fig. S5c). This can be explained by the strong influence of Pt Schottky contact on thin NWs, which generates a strong built-in electric field that effectively modulates the electron concentration at the NW surface, facilitating the O_2 ionization and acetone reduction process.

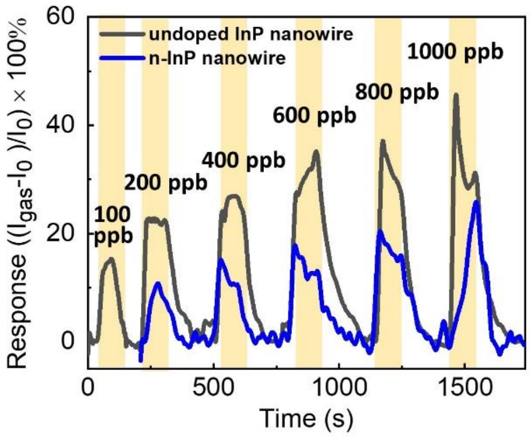

Figure S6. The time-dependent acetone sensing measurement with the chitosan modified InP NW sensors which are undoped and n-doped sensor at zero bias, respectively. The device fabricated from n-doped InP NW (doping concentration ~3×10^{18} cm^{-3}) showed a decreased response compared to the undoped InP NW sample due to the larger baseline current.

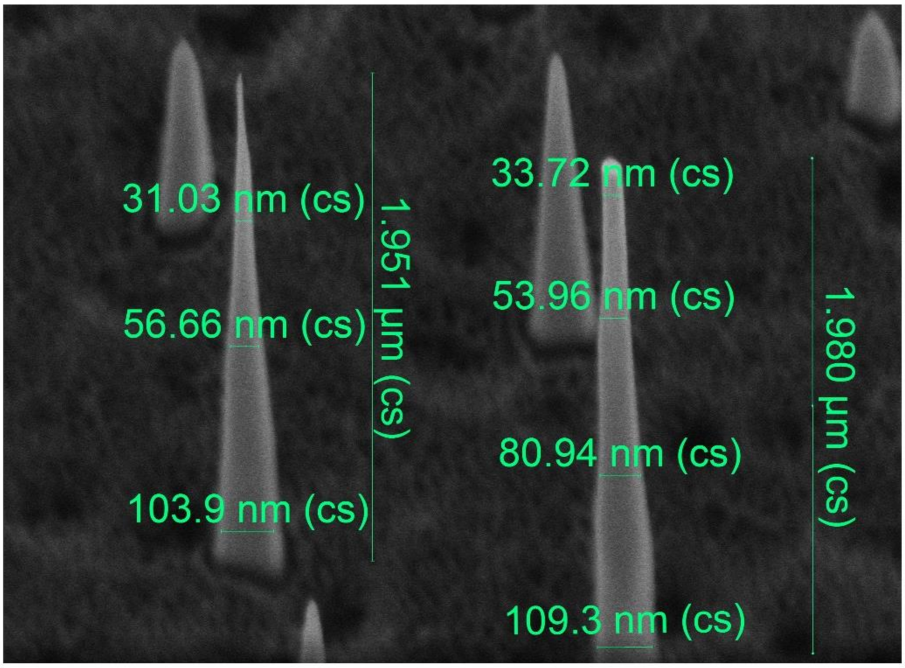

Figure S7. The SEM image of the top-down etched InP NWs with the diameter and length measurements. The NWs exhibit slightly different morphology, i.e., size, shape and surface roughness from the bottom-up grown NWs shown in Fig. S1.

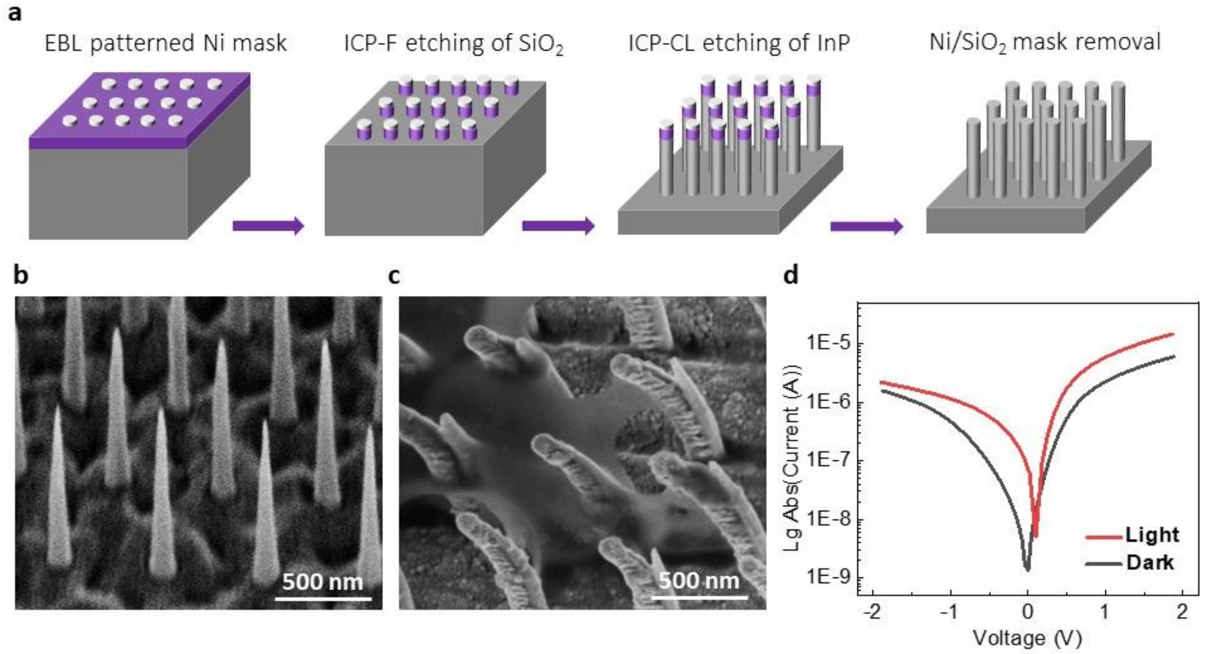

Figure S8. The top-down etched NW array and device (t-InP/Pt/chitosan) fabrication and electric measurement. **a,** Schematics of top-down etching approach for InP NW array fabrication: EBL patterned Ni (thickness 70 nm, diameter 40 nm) mask on PECVD deposited SiO_2 (thickness 600 nm), which was pre-deposited on undoped InP wafer ((1-10) × 10^{15} cm^{-3}); ICP-F etching of SiO_2 layer to form the SiO_2/Ni mask; ICP-CL etching of the InP wafer with the SiO_2/Ni mask; SiO_2/Ni mask removal by 10% HF solution. SEM images of **b**, InP NWs after ICP-CL etching, and **c**, after Pt and chitosan deposition. **d**, I-V measurement of the t-InP/Pt/chitosan NW sensor under the dark/light condition with solar simulator @AM 1.5, 100 mW/cm^2.

Due to the small and non-uniform NW diameter and possible top-down etching-induced surface damage, the top-down NW device exhibits a weaker photovoltaic effect with an I_{SC} of 61 nA and a V_{OC} of 99 mV. Nevertheless, it is sufficient for self-powered operation, producing a selective, stable, and fast response highly desirable for breath testing.

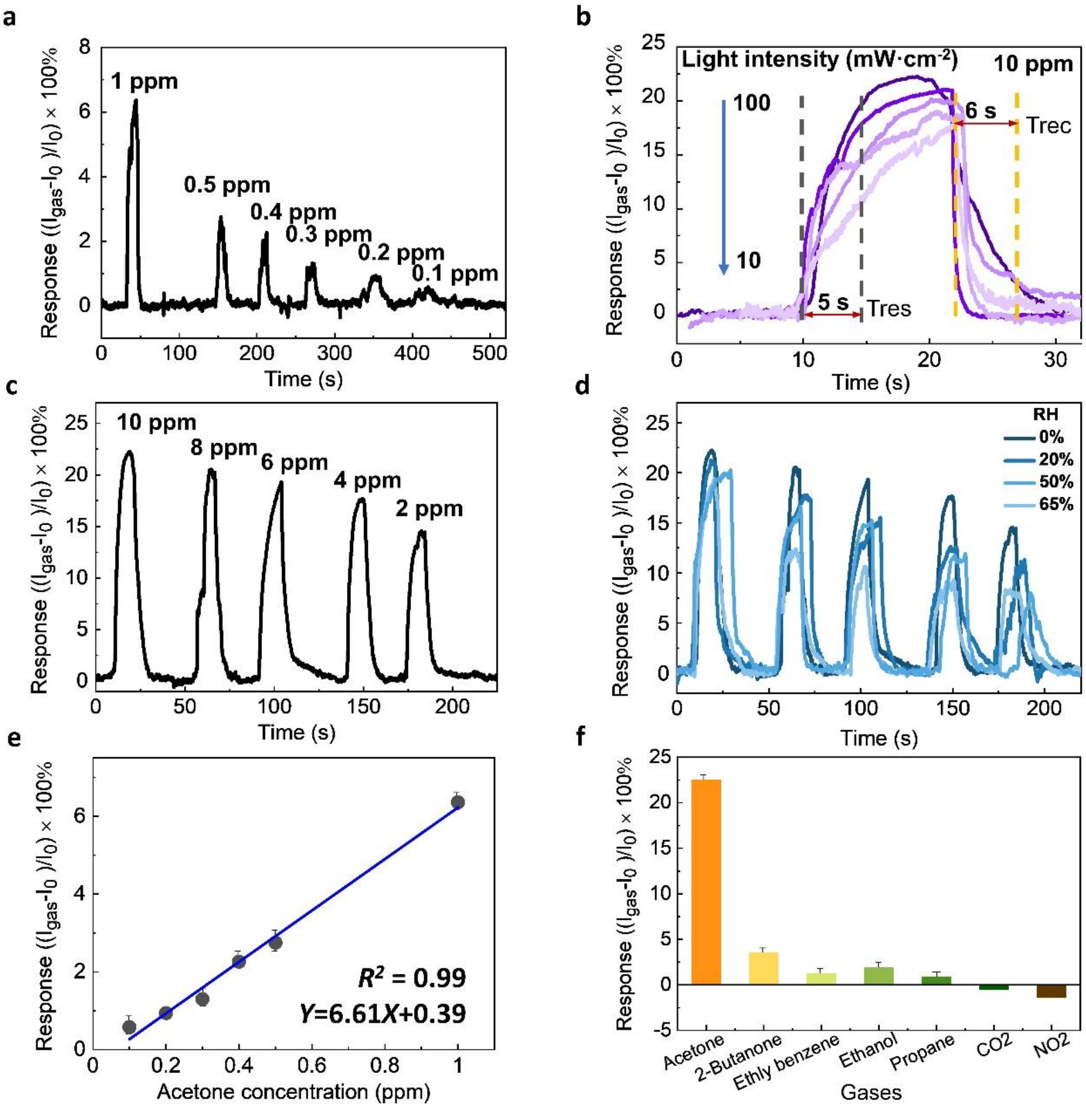

Figure S9. Acetone sensing performance of the top-down method fabricated NW sensor (t-InP/Pt/chitosan). **a**, Time-dependent sensing response measured for an acetone concentration of 0.1-1 ppm. **b**, Acetone sensing response under different light illumination intensities from 100 to 10 mW·cm^{-2}, with the response and recovery times of the response curve under 100 mW·cm^{-2} light illumination being indicated. **c**, Time-dependent sensing response curve with an acetone concentration range of 2-10 ppm. **d**, Time-dependent sensing response to the acetone concentration of 2-10 ppm under the relative humidity (RH) levels of 0%, 20%, 50%, 65%. **e**, Sensor response vs concentration curve with linear fitting. **f**, Gas sensing selectivity measurement to compare the response to 10 ppm acetone, 2-butanone, ethyl benzene, ethanol, propane, NO_2, and 10% CO_2. The error bars in **e**, **f** indicate the standard deviation obtained from 10 cycles of sensing measurements.

The performance comparison between b-InP/Pt/chitosan and t-InP/Pt/chitosan NW acetone sensor is summarized in Table S2, indicating that the bottom-up NW sensor is more sensitive

than the top-down sensor, whereas the latter has a faster response speed. This may be ascribed to the less ideal NW morphology due to top-down etching, i.e., the tapered and bent NWs with a rough surface. Such morphology causes less chitosan coverage and slightly degraded electrical properties. Nevertheless, the performance of the top-down NW sensor is adequate for breath acetone sensing, as confirmed by the successful demonstration of the Ketowhistle prototype; in particular, the fast response speed is highly desirable for breath-sensing applications.

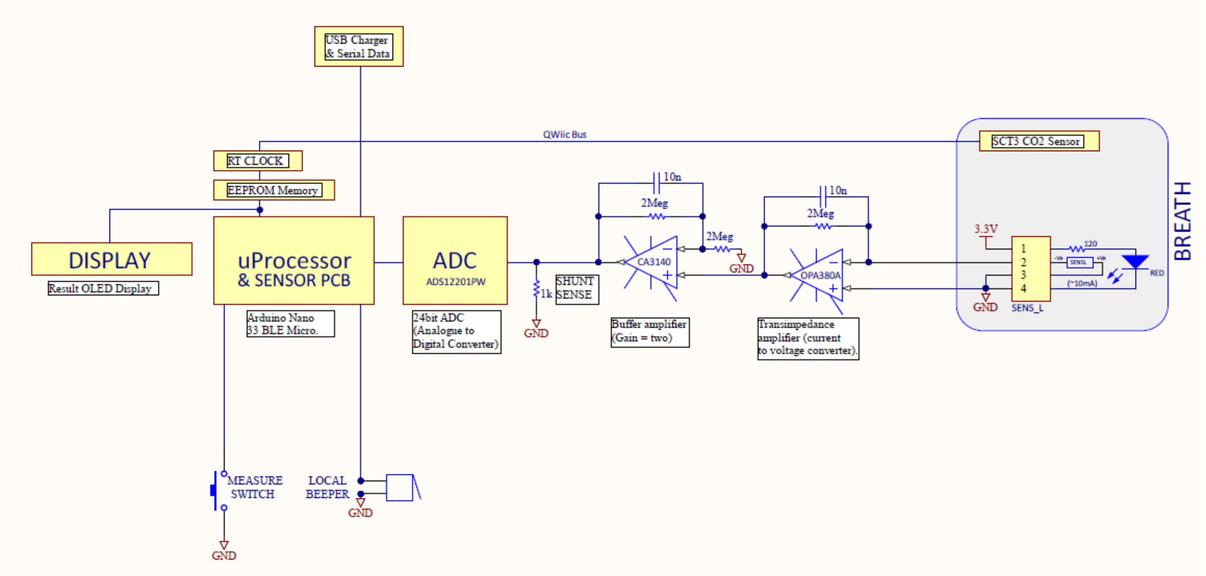

Figure S10. Block diagram of the electrical circuit in the Ketowhistle. The acetone sensor is located at the “Breath” frame and powered by a low-power red LED. The output signal is captured by measuring the voltage of a 2M load resistor in series with the amplified sensor current signal. The actual current from the acetone sensor can be calculated as: current = output voltage / (4 × 10^6 Ω).

Table S3. The parameters of the electrical elements in the block diagram shown in Figure S10.

Comment	Description
Op Amp (CA3140)	FET Operational Amplifier (Gain = two)
Op Amp (OPA380A)	Transimpedance amplifier (current to voltage converter)
Cap	Capacitor
Red	Typical Infrared GaAs LED (Digikey, LS R976-NR-1), power = 3.2 mW, peak wavelength = 645 nm
Beeper	Magnetic Transducer Buzzer
SWITCH	Switch
SENS_L	Header, 4-Pin
Res1	Resistor 2M ohm
SENSL	Resistor

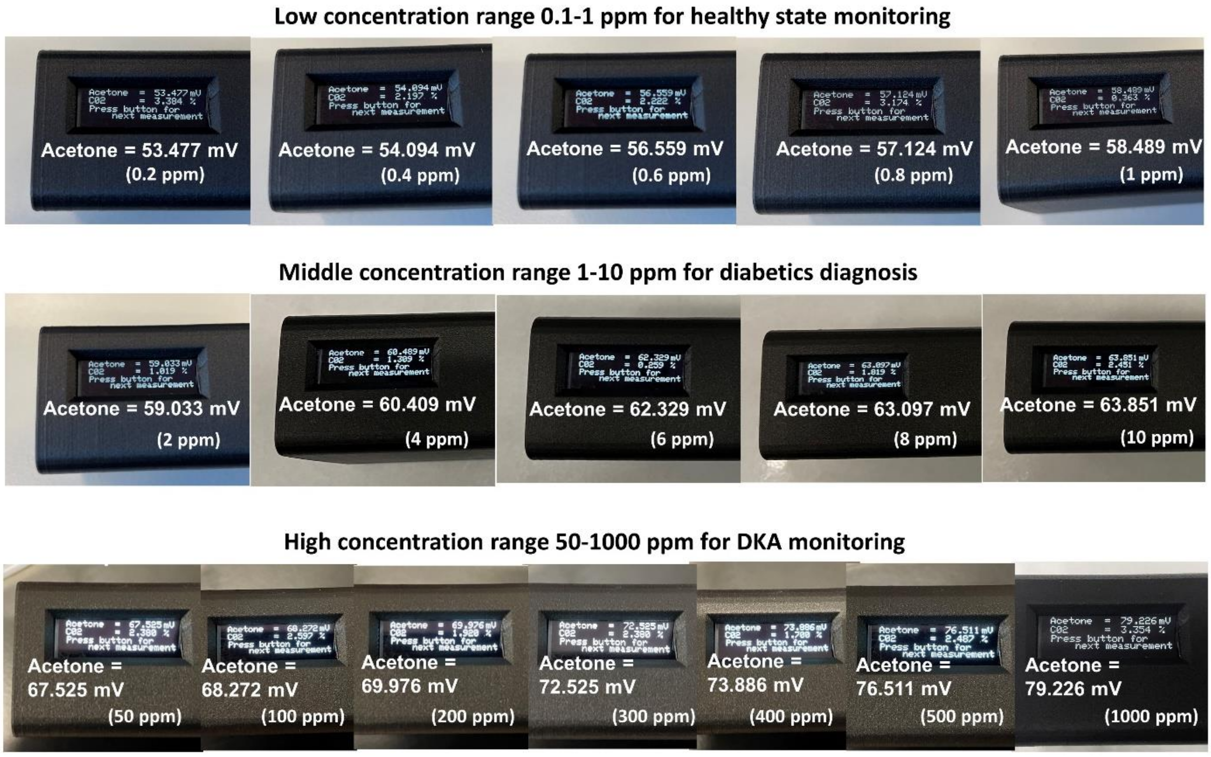

Figure S11. Recorded pictures for ketone whistle calibration for low (0.1 – 1 ppm), medium (1 – 10 ppm), and high (50 - 1000 ppm) acetone concentration range, respectively.

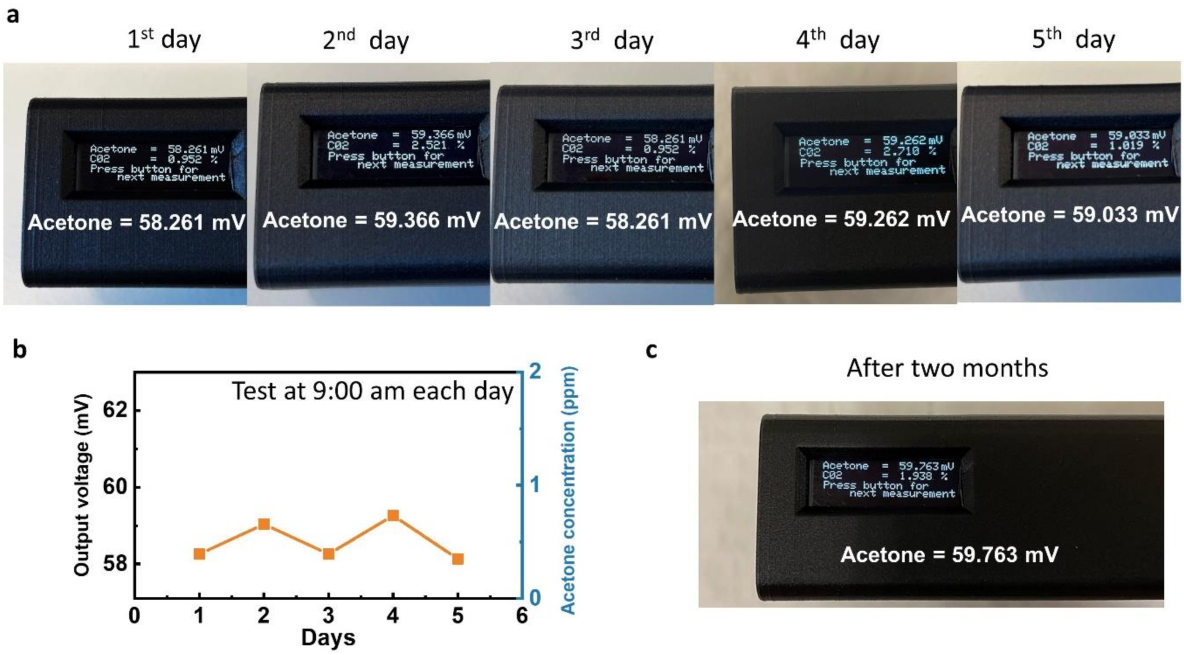

Figure S12. **a**, The Ketowhistle breath testing result recording from the same person at 9:00 am for five consecutive days and **b**, the corresponding acetone concentration based on the calibration data presented in Fig. 5c. **c**, The Ketowhistle breath testing result recorded from the same person after two months with the Ketowhistle stored in the ambient condition.

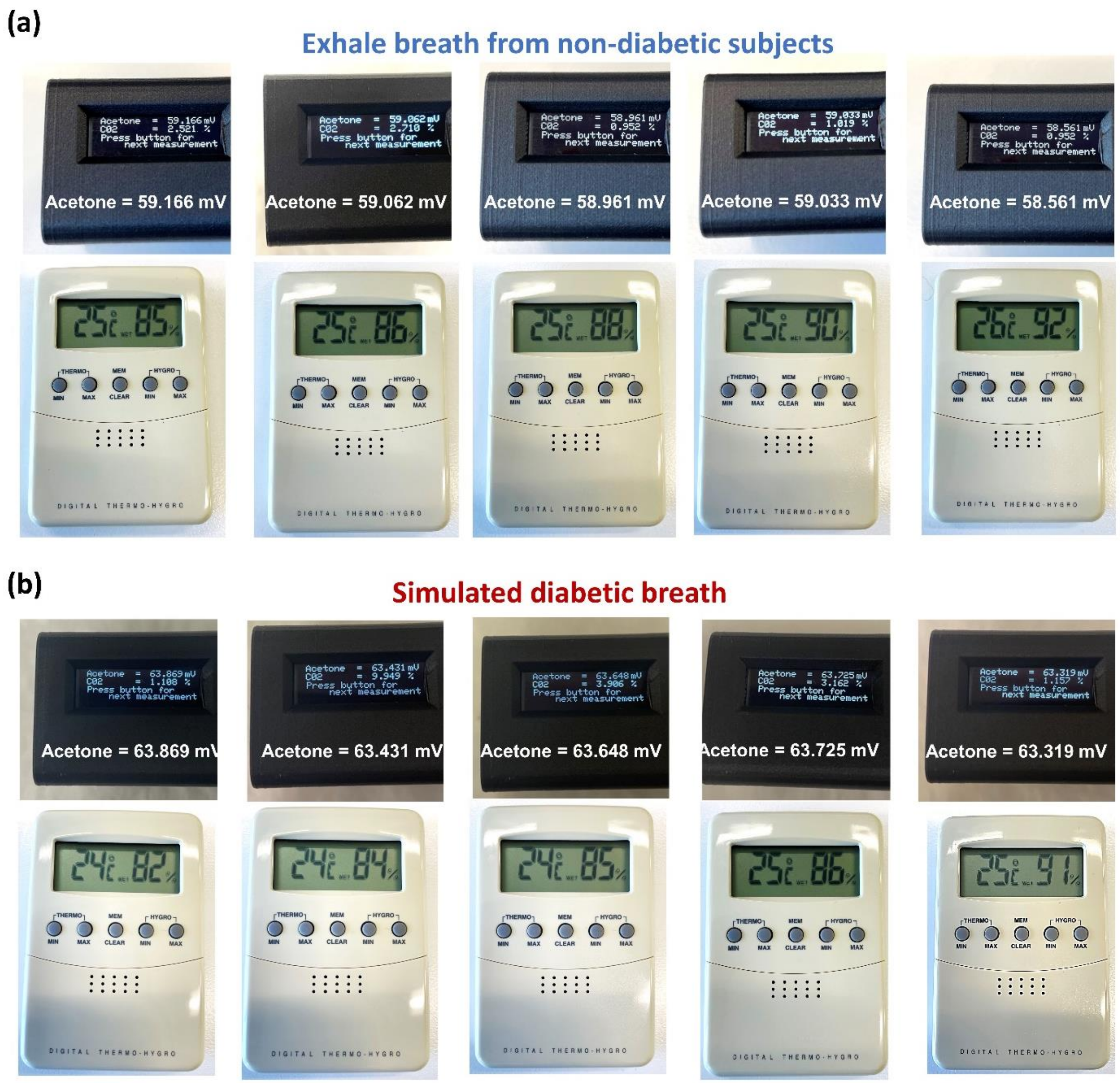

Figure S13. The Ketowhistle breath testing result breath samples from non-diabetic subjects (a) and simulated diabetic breath samples (b). The RH of the breath sample was measured along with acetone by a digital RH sensor and shown under each Ketowhistle measurement.

Reference

[1] N. Kaur, D. Zappa, V. A. Maraloiu,E. Comini, *Adv. Funct. Mater.* **2021**, 31, 2104416.
[2] S. J. Choi, I. Lee, B. H. Jang, D. Y. Youn, W. H. Ryu, C. O. Park,I. D. Kim, *Anal. Chem.* **2013**, 85, 1792.
[3] I. C. Weber, H. P. Braun, F. Krumeich, A. T. Guntner,S. E. Pratsinis, *Adv. Sci.* **2020**, 7, 2001503.
[4] X. Y. Kou, N. Xie, F. Chen, T. S. Wang, L. L. Guo, C. Wang, Q. J. Wang, J. Ma, Y. F. Sun, H. Zhang,G. Y. Lu, *Sens. Actuators B Chem.* **2018**, 256, 861.

[5] Y. J. Jeong, W. T. Koo, J. S. Jang, D. H. Kim, H. J. Cho,I. D. Kim, *Nanoscale* **2018**, 10, 13713.
[6] Q. A. Drmosh, Y. A. Al Wajih, I. O. Alade, A. K. Mohamedkhair, M. Qamar, A. S. Hakeem,Z. H. Yamani, *Sens. Actuators B Chem.* **2021**, 338, 129851.
[7] R. Jaisutti, M. Lee, J. Kim, S. Choi, T. J. Ha, J. Kim, H. Kim, S. K. Park,Y. H. Kim, *ACS Appl Mater Interfaces* **2017**, 9, 8796.
[8] F. D. Qu, Y. Yuan, R. Guarecuco,M. H. Yang, *Small* **2016**, 12, 3128.
[9] Z. R. Zhang, Y. X. Wu, H. Y. Du, Y. H. Sun, S. P. Sun, S. K. Xu, L. Y. Cong,P. C. Sun, *J. Alloys Compd.* **2022**, 895, 162017.
[10] M. Y. Chuang, Y. T. Lin, T. W. Tung, L. Y. Chang, H. W. Zan, H. F. Meng, C. J. Lu,Y. T. Tao, *Sens. Actuators B Chem.* **2018**, 260, 593.
[11] T. I. Nasution, I. Nainggolan, S. D. Hutagalung, K. R. Ahmad,Z. A. Ahmad, *Sens. Actuators B Chem.* **2013**, 177, 522.
